# Tumor necrosis factor-α-induced protein 8-like 2 (TIPE2) is associated with immune phases of patients with chronic hepatitis B

**DOI:** 10.18632/oncotarget.15683

**Published:** 2017-02-24

**Authors:** Yu-Chen Fan, Yuan-Yuan Zhang, Na Wang, Yan-Yan Sun, Kai Wang

**Affiliations:** ^1^ Department of Hepatology, Qilu Hospital of Shandong University, Jinan, China; ^2^ Institute of Hepatology, Shandong University, Jinan, China; ^3^ Department of Neurology, Jinan Central Hospital Affiliated to Shandong University, Jinan, China

**Keywords:** TIPE2, immune phase, chronic hepatitis B, immune clearance, interleukin, Immunology and Microbiology Section, Immune response, Immunity

## Abstract

Tumor necrosis factor-α-induced protein 8-like 2 (TIPE2) is a newly negative immune regulator but its role in different immune phases of patients with chronic hepatitis B (CHB) is unknown. We determined the mRNA levels of TIPE2, interleukin-6, interleukin-10, tumor necrosis factors-α and interferon-γ in peripheral blood mononuclear cells from 205 naïve treated CHB patients and 15 healthy controls by quantitative real time polymerase chain reaction. Intrahepatic TIPE2 protein was also determined using immunohistochemistry staining. The TIPE2 mRNA level in CHB patients was significantly higher than that in healthy controls. Moreover, the TIPE2 mRNA level in immune clearance (IC) phases was significantly higher than that in immune tolerance (IT) phase; whereas TIPE2 mRNA in HBeAg negative hepatitis (ENH) was obviously higher than low replication (LR) phase. Furthermore, the optional cut off values of 2.02 and 1.59 for TIPE2 mRNA level have strong power in identifying IC and ENH from IT and LR. In addition, intrahepatic TIPE2 protein was predominantly located in hepatocyte plasma and correlated with hepatic inflammatory and fibrosis. Multivariate analysis showed tumor necrosis factors-α, interferon-γ and HBV DNA load were independently correlated with TIPE2 level. In conclusion, TIPE2 might be associated to the immune clearance of patients with chronic hepatitis B.

## INTRODUCTION

Hepatitis B virus (HBV) is a serious public problem and about more than 350 million subjects are HBV carriers globally [1]. Immune response is recognized as one of the main determinants for the progression of chronic HBV infection [2]. Due to the characteristics of immune statue, the natural course of patients with chronic hepatitis B (CHB) can be typically divided into four phases: immune tolerance (IT), immune clearance (IC), low or no-replicative (LR), and hepatitis B e antigen (HBeAg)-negative hepatitis (ENH) [1, 3–6]. Understanding the natural course of CHB makes a lot of sense on exploring the progression of this disease, searching for potential therapeutic target, and optimizing antiviral treatment [7, 8]. However, the exact function and mechanism of immune regulation in chronic hepatitis B remains still obscure.

Tumor necrosis factor-α-induced protein 8-like 2(TIPE2) is a novel molecular belonging to TNFAIP8 family, and orchestrates immune homeostasis by binding to T cell receptors and toll-like receptors [9]. TIPE2 can promote Fas-mediated apoptosis and stimulate the activation of cell death [9, 10]. Previous studies demonstrated that TIPE2 knockout cells exert strong biological function stimulated by activating toll-like receptor and T cell receptor signals pathway [11]. Furthermore, the levels of cytokines including interleukin(IL)-1, IL-6, IL-12, and tumor necrosis factor (TNF)-α as well as the inhibitory cytokine IL-10 were significantly produced in TIPE2-deficient mice [9]. TIPE2 has also been reported to regulate innate immunity to bacteria and dsRNA viruses by targeting the Rac GTPase, and down-regulation of TIPE2 is associated with increased phagocytosis and bacterial killing [12, 13]. Accelerating evidences support the hypothesis that TIPE2 might contribute to the pathogenesis in a variety of chronic inflammatory diseases, autoimmune disorders, stroke, diabetic nephropathy, tumors and atherosclerosis [14–24]. Recent studies demonstrated that TIPE2 production was inhibited in HBV specific T cell response and significantly correlated with the grades of hepatic inflammation in the patients with viral hepatitis [11, 14]. T cell immunity exerts dual roles in the clearance of HBV replication and contributes to the progression of chronic HBV infection [25]. Therefore, TIPE2 might participate in the immune phases of patients with chronic hepatitis B.

Currently, we have previously revealed that TIPE2 contributed to the occurrence of liver failure and peripheral TIPE2 mRNA level might be a biomarker for predicting the 3-month mortality of liver failure [26, 27]. However, the potential role of TIPE2 in the various immune phases of chronic hepatitis B remains still unknown. This present study was to assess the expression of TIPE2 in the natural history of chronic hepatitis B with different immune phases. Here, we first determined the mRNA expression levels of TIPE2, IL-6, IL-10, TNF-α, and IFN-γ in peripheral blood mononuclear cells from 205 naïve treated patients with chronic hepatitis B, as well as 15 healthy controls. Second, we also determined the location and expression of intrahepatic TIPE2 protein in liver tissue. Furthermore, we investigated the possible diagnosis value of TIPE2 mRNA in discriminating different immune stages of CHB patients if necessary.

## RESULTS

### General characteristics

**Figure 1:**
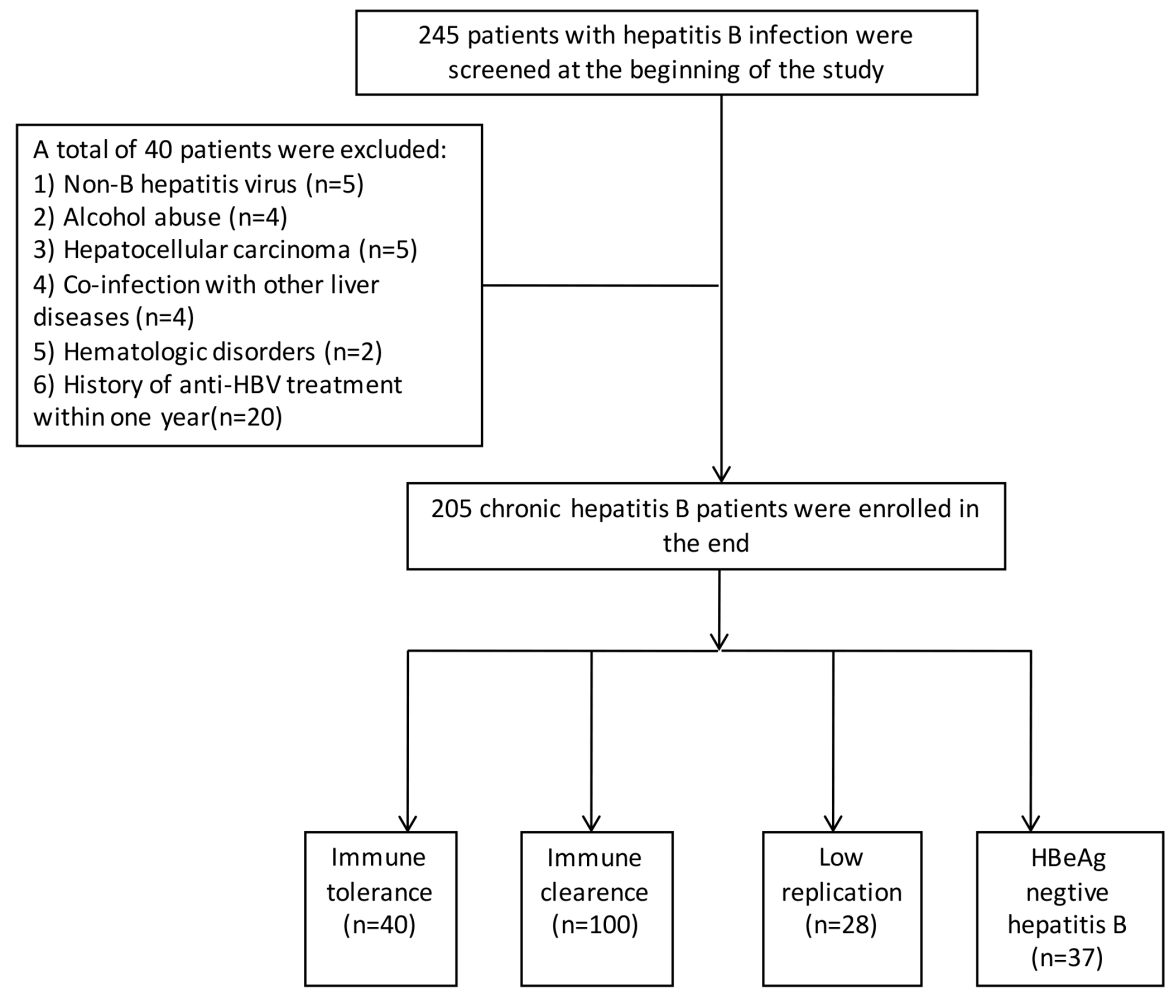
Flowchart for the inclusive procession of all the subjects in this present study

Figure 1 was shown for describing the inclusion of patients in our present study. From Dec 2013 to Jan 2015, a total of 245 patients with HBsAg positive for more than 6 months were hospitalized and retrospective collected. Of whom, a total of 40 patients were excluded as the following reasons: 5 for non-B hepatitis virus infection, 4 for severe alcohol abuse, 5 for hepatocellular carcinoma, 4 for co-infection with other liver diseases, 2 for hematologic disorders, and 20 for the history of anti-HBV treatment within the near one year. Finally, a total of 205 naïve CHB patients were included and were divided into four groups: 40 patients for IT phase, 100 for IC phase, 28 for LR phase, and 37 for ENH phase. The general characteristics of CHB patients and healthy controls were shown in Table 1.

**Table 1:** Baseline characteristics of the enrolled patients

Variables	CHB group (*n* = 205)	HC group (*n* = 15)	*P* Values
Male (n, %)	152(74.1)	11(73.3)	0.945a
Age (years)	40(28-47)	38(26-45)	>0.05b
HBeAg+(n, %)	140(68.3)	NA	
HBVDNA+(n, %)	164(80.0)	NA	
Log10 (HBVDNA load)	5.74(3.92-7.22)	NA	
HBsAg(IU/mL)	3769(994.5-5711)	NA	
ALT (U/L)	120(39-265.5)	16(12.0-25.0)	<0.001^b^
AST (U/L)	57(32.5-132)	12(7.0-20.5)	<0.001^b^
TBIL (umol/L)	17.7(11.7-41.7)	10.5(6.7-12.3)	<0.001^b^
ALB (g/L)	42(37.7-45.1)	50(42-61)	<0.001^b^
PT-INR	1.00(0.95-1.06)	0.80(0.72-1.01)	0.688^b^
PTA (%)	92(77.5-102)	89(78-93)	0.237^b^
AFP (ng/ml)	29.6(13.2-41.2)	25(19-31)	<0.001b
Cr (umol/L)	62(52-69.6)	63(52-69)	0.282b
WBC (E+09/L)	5.40(4.60-6.69)	7.65(6.32-8.65)	<0.001b
HGB (g/L)	125(110-145.5)	143(130-156)	<0.001^b^
PLT (E+09/L)	187(154-225.5)	201(157-234)	0.006^b^

Quantitative variables were expressed as the median (centile 25; centile 75).

Categorical variables were expressed as number (%).

### The mRNA levels of TIPE2 and its associated cytokines in peripheral blood mononuclear cells from CHB patients and healthy controls

**Figure 2:**
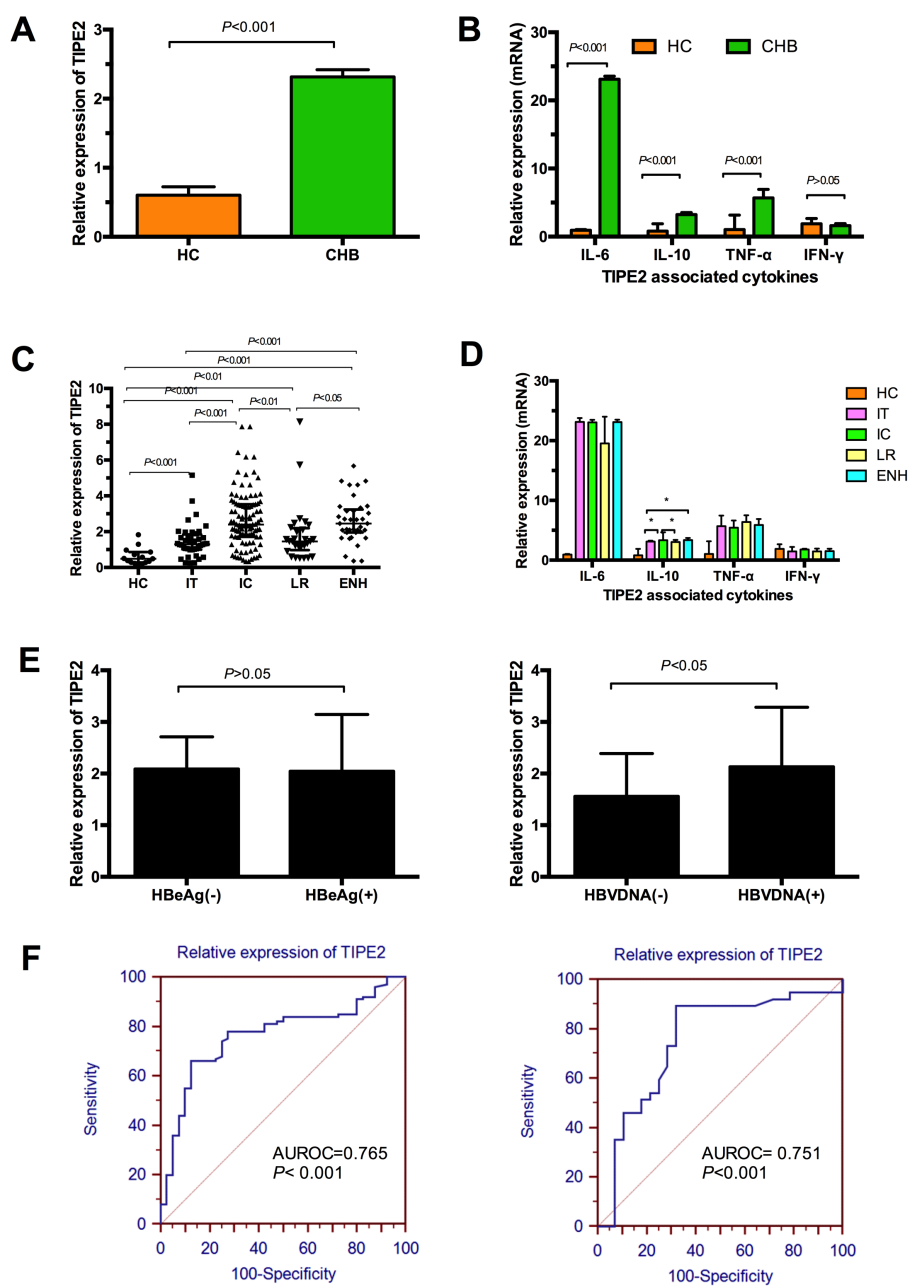
Relative expression of TIPE2 mRNA and TIPE2 associated cytokines in peripheral blood mononuclear cells in different immune phases of chronic hepatitis and healthy controls **A**. The relative expression of TIPE2 mRNA in peripheral blood mononuclear cells in all the patients and healthy controls. **B**. The mRNA expression levels of IL-6, IL-10, TNF-α, and IFN-γ in all the patients and healthy controls. **C**. Expression of TIPE2 mRNA from peripheral blood mononuclear cells in healthy controls and CHB patients with IT, IC, LR, and ENH. **D**. The mRNA expression levels of IL-6, IL-10, TNF-α, and IFN-γ in healthy controls and CHB patients with IT, IC, LR and ENH. **E**. Expression of TIPE2 mRNA in CHB patients stratified by the statue of HBeAg and HBVDNA. F. ROC analysis of the predicative accuracy for TIPE2 mRNA in discriminating IC from IT and ENH from LR.

In this present study, we first compared the differences of TIPE2 and its associated cytokines between CHB patients and healthy controls. Figure 2A illustrated that the relative expression of TIPE2 mRNA in CHB patients was significantly higher than that in healthy controls (CHB: 2.061 [1.285, 3.08] vs. 0.49 [0.28, 0.86], *P* < 0.01), indicating TIPE2 might participate in the progression of HBV infection. Then we have determined the relative mRNA levels of TIPE2 associated cytokines including IL-6, IL-10, TNF-α, and IFN-γ in CHB patients and healthy controls. As shown in Figure 2B, we demonstrated that the mRNA expression levels of IL-6, TNF-α and IL-10 in CHB patients were significantly increased compared with healthy controls (IL-6, 23.12 [4.4, 23.57] vs. 0.93 [0.72, 1.05], *P* < 0.01; TNF-α, 5.68 [4.62, 6.90] vs. 1.04 [0.63, 3.17], *P* < 0.01; IL-10, 3.24 [2.95, 3.54] vs. 0.49 [0.28, 0.86], *P* < 0.01), whereas we did not find any significant differences of IFN-γ between the two groups (1.61 [1.21, 1.92] vs. 1.90 [0.92, 2.66], *P*> 0.05).

### Expression of TIPE2 mRNA from peripheral blood mononuclear cells and TIPE2 associated cytokines in the different immune phases of CHB patients

The general characteristics of CHB patients stratified by the different immune phases were shown in Table 2. The median and interquartile ranges for the relative mRNA expression of TIPE2 were 1.3 [0.997, 1.847] for IT patients, 2.396 [1.712, 3.53] for IC patients, 1.45 [0.976, 2.197] for LR patients, and 2.45 [1.95, 3.24] for ENH patients. In Figure 2C, we demonstrated that the relative expression of TIPE2 mRNA in each of the four immune phases was significantly higher than healthy controls (0.49 [0.28, 0.86]). Furthermore, we found that relative expression of TIPE2 mRNA in IC/ENH phases shared similar higher trend for TIPE2 mRNA level compared with those with LR/IT phases (all *P* < 0.05, respectively). Furthermore, we did not find any significant difference of TIPE2 mRNA in ENH and IC phases (*P*>0.05), as well as LR and IT phases (*P*>0.05). Current evidences suggested that immune response contribute to progression from IT to IC, and LR to ENH, and then leading to virus elimination. Therefore, we investigated the differences of TIPE2 associated cytokines in the four immune phases. Figure 2D revealed that the level of IL-10 in IC group (3.33 [3.12, 4.65]) was significantly higher than that in LR group (3.03 [2.79, 3.39], *P* < 0.05) or IT group (3.11 [0.31, 3.27], *P* < 0.05), whereas we also demonstrated the significant difference of IL-10 between IT group and ENH group (3.11 [0.31, 3.27] vs. 3.37 [3.08, 3.71], *P* < 0.05). However, we did not find any significant differences of IL-6, TNF-α and IFN-γ between any two of the four phases.

**Table 2:** Baseline characteristics of immune phases of chronic hepatitis B

Variable	HBeAg (+)		HBeAg(-)		*P*
	IT (*n* = 40)	IC (*n* = 100)	LR (*n* = 28)	ENH (*n* = 37)	
Male (%)	25(62.5)	78 (78.0)	19 (67.8)	28 (75.7)	0.554^a^
Age (years)	32(24-39)	38 (28-46)	41(26-51)	40(32-54)	<0.001^b^
HBeAg+(%)	40 (100)	100 (100)	0 (0)	0 (0)	<0.001^a^
HBVDNA+(%)	40 (100)	100 (100)	7(25.0)	35(94.6)	<0.001^a^
Log10 (HBVDNA load)	7.70 (7.25-8.18)	5.07 (4.75-6.73)	0 (0-3.19)	5.30 (4.04-6.04)	<0.001^b^
HBsAg(IU/mL)	4489(3595-6512)	2332(838-5407)	1956(212-3775)	2905(1770-4161)	<0.001^b^
ALT (U/L)	29 (21-39)	263 (1367-453)	35 (24-41)	253 (123-465)	<0.001^b^
AST (U/L)	27 (22-33)	117 (67-232)	20 (18-36)	152(98-319)	<0.001^b^
TBIL (umol/L)	13.1 (7.4-18.6)	49.8 (18.4-87.1)	12.5 (9.5-17.7)	18 (15-25)	<0.001^b^
ALB (g/L)	43.6 (42.3-48.3)	41.8 (39.1-44.7)	43.2 (40.1-46.2)	44.8 (40.8-47.9)	<0.001^b^

Quantitative variables were expressed as the median (centile 25; centile 75). Categorical variables were expressed as number (%).

a, Chi-square test; b, Kruskal-Wallis test.

IT, immune tolerance phase; IC, immune clearance phase; LR, low-replicative phase; ENH, HBeAg-negative hepatitis phase; HBeAg, hepatitis B e antigen; HBsAg, hepatitis B surface antigen;ALT, alanine aminotransferase; AST, aspartate aminotransferase; TBIL, total bilirubin; ALB, albumin.

When the serum viral load of HBV DNA is less than 500 IU/mL, it is recognized as negative.

To explore the possible relationship of TIPE2 with HBV replication, we also compared the TIPE2 mRNA levels in the CHB patients stratified by the statue of HBeAg and HBVDNA as shown in Figure 2E. No significant difference was found between CHB patients with HBeAg positive (IT+IC) and those without HBeAg positive (LR+ENH) (2.091 [1.395, 2.711] vs.2.045 [1.28, 3.145], *P* > 0.05). Of note, we demonstrated a significant higher level of TIPE2 mRNA in HBVDNA positive patients compared with HBVDNA negative patients (2.131 [1.382, 3.285] vs. 1.56 [0.870, 2.391], *P* < 0.05). Furthermore, the receiver operating characteristic (ROC) analysis was performed to identify whether TIPE2 mRNA could discriminate IC phase from IT phase, and ENH phase from LR phase. Figure 2F showed the area under the receiver operating characteristic (AUROC) curves of TIPE2 mRNA for predicting the incidence of IC in the IT individuals was 0.765 (95% confidence interval 0.686-0.832, *P* < 0.001), and the optimal cutoff value was 2.02 with a sensitivity of 66.00% and a specificity of 87.5%. Meanwhile, the AUROC of TIPE2 mRNA for the incidence of ENH in the LR individuals was 0.751 (95% confidence interval 0.628-0.850, *P* < 0.001), and the optimal cutoff value was 1.59 with a sensitivity of 89.19% and a specificity of 67.86%.

### Intrahepatic TIPE2 expression was associated with inflammation and fibrosis in CHB patients

**Figure 3:**
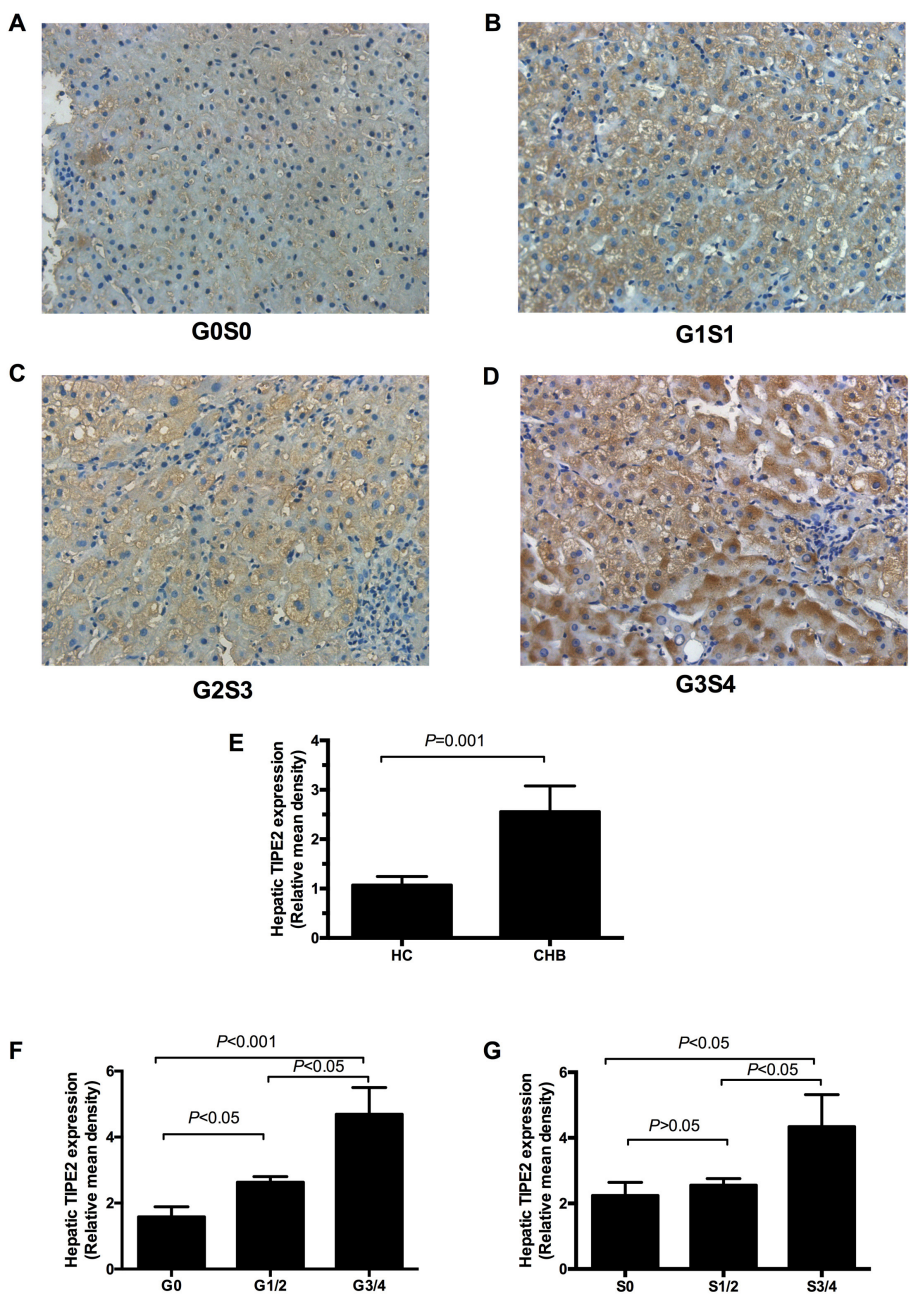
Expression of TIPE2 protein in liver tissue of CHB patients **A**.-**D**. The positive staining for TIPE2 was found mainly in the hepatocytes (200×). **E**. Relative mean density analysis showed the difference in hepatic TIPE2 staining between CHB group and healthy controls. **F**. Expression of hepatic TIPE2 staining between inflammation grade 0, grade (1-2) and grade (3-4). **G**. Expression of hepatic TIPE2 staining between fibrosis stage (3-4), stage (1-2), and fibrosis stage 0.

We also determined the expression of intrahepatic TIPE2 protein from 25 CHB patients and 4 normal livers from liver transplant donors (Table 3). Representative immunohistochemical images of TIPE2 in CHB patients were shown in Figure 3A-3D. The local TIPE2 protein was highly stained and mainly visualized in hepatocyte cytoplasm. Digital image analysis showed that there were significant differences of relative mean integrated optical density for hepatic TIPE2 in CHB patients and normal livers (*P* < 0.05) in Figure 3E. Furthermore, we also found that the relative mean integrated optical densities for hepatic TIPE2 protein in inflammation G3/4 and fibrosis S3/4 were significantly higher compared to that of the inflammation G1/2 and fibrosis S1/2 (*P* < 0.05, Figure 3F). Importantly, we also demonstrated that the relative mean density of TIPE2 expression was significantly increased in CHB patients with G1/2 than those with G0 (*P* < 0.05). There was no significant difference of the relative mean integrated optical density for hepatic TIPE2 protein between fibrosis S0 and S 1/2 (*P* > 0.05).

**Table 3:** Baseline characteristics of the CHB patients receiving a liver biopsy

Cases	Gender	Age	G (Inflammation)	S (Fibrosis)	ALT (U/L)	AST (U/L)	HBeAg (+/−)	HBVDNA (+/−)	Immune phase
1	Female	29	0	0	16	26	-	+	IT
2	Female	24	0	0	123	72	+	+	IC
3	Male	40	0	1	49	47	+	+	IC
4	Male	38	0	1	116	41	+	+	IC
5	Female	56	0	1	23	24	-	-	LR
6	Female	27	1	0	10	17	-	+	LR
7	Male	29	1	0	67	45	+	+	IC
8	Male	46	1	0	54	47	-	+	ENH
9	Male	38	1	1	109	87	+	+	IC
10	Female	35	1	1	97	48	-	+	ENH
11	Female	31	1	0	11	21	+	+	IT
12	Male	29	1	1	56	35	+	+	IC
13	Male	40	0	2	21	25	+	+	IT
14	Male	47	1	2	102	77	+	-	ENH
15	Female	19	2	0	23	42	+	+	IT
16	Male	40	2	1	18	18	+	-	LR
17	Male	31	2	2	155	74	+	+	IC
18	Male	36	2	1	262	94	+	+	IC
19	Male	23	3	2	225	110	+	+	IC
20	Female	41	3	3	89	62	+	+	IC
21	Male	20	2	3	107	61	-	-	ENH
22	Female	47	3	4	310	105	+	+	IC
23	Male	31	4	3	166	121	+	+	IC
24	Female	47	2	4	132	105	+	+	IC
25	Male	39	4	3	176	102	+	+	IC
Control1	Male	39	0	0	22	35	-	-	NA
Control2	Male	34	0	0	31	28	-	-	NA
Control3	Male	41	0	0	18	29	-	-	NA
Control4	Female	28	0	0	27	21	-	-	NA

IT, immune tolerance phase; IC, immune clearance phase; LR, low-replicative phase; ENH, HBeAg-negative hepatitis phase; HBeAg, hepatitis B e antigen; ALT, alanine aminotransferase; AST, aspartate aminotransferase;

### Correlations between TIPE2 mRNA levels and clinical characteristics in CHB patients

We performed univariate and multivariate analysis to investigate the possible associations of TIPE2 mRNA with clinical characteristics in CHB patients. In Table 4, the relative expression of TIPE2 mRNA was significantly positively correlated with HBV DNA load (r = 0.200, *P* < 0.001), alanine aminotransferase (ALT) (r = 0.300, P < 0.001), aspartate aminotransferase (AST) (r = 0.283, *P* < 0.001), and IL-10 (r = 0.147, *P* < 0.05); whereas TIPE2 mRNA was negatively correlated with PT-INR (r = -0.151, *P* < 0.001), IL-6(r = -0.163, *P* < 0.05), TNF-α (r = -0.159, *P* < 0.05), and IFN-γ (r = -0.183, *P* < 0.01). Multivariate analysis showed that three independent variables were associated with TIPE2 mRNA: HBV DNA load (β = 0.196, *P* < 0.05), TNF-α (β = -0.142, *P* < 0.05), and IFN-γ (β = -0.151, *P* < 0.05).

**Table 4:** Correlations between TIPE2 mRNA levels and other parameters using univariate and multivariate analysis in all the patients and healthy controls

TIPE2 mRNA vs.	Univariate analysis		Multivariate analysis	
	R	*P*	β	*P*
Gender	0.120	0.087		
Age (years)	-0.094	0.180		
HBeAg(IU/mL)	0.018	0.795		
HBVDNA	0.200	0.004	0.196	0.007
HBsAg(IU/mL)	0.081	0.250		
ALT (U/L)	0.300	0.000	-0.027	0.857
AST (U/L)	0.283	0.000	0.063	0.673
TBIL (umol/L)	0.106	0.129		
ALB (g/L)	0.032	0.646		
PT-INR	-0.151	0.031	0.026	0.714
WBC (E+09/L)	0.044	0.535		
HGB (g/L)	-0.005	0.942		
PLT (E+09/L)	-0.002	0.977		
IL-6 (pg/ml)	-0.163	0.020	-0.035	0.624
IL-10(pg/ml)	0.147	0.036	0.098	0.177
TNF-α(pg/ml)	-0.159	0.023	-0.142	0.044
IFN-γ (pg/ml)	-0.183	0.009	-0.151	0.032

## DISCUSSION

TIPE2 is a novel identified negative regulator in immune homeostasis but its role in the natural history of chronic HBV infection has not been well investigated. In this present study, we determined the dynamic expression of TIPE2 mRNA in PBMCs in different immune phases of chronic HBV infection. To our knowledge, this is the first report to investigate the relationship between TIPE2 and immune phases in the natural history of chronic HBV infection.

Firstly, we demonstrated that the relative level of TIPE2 mRNA in CHB patients was significantly higher than healthy controls. These results are in disagreement with the previous report [14]. The discrepancy of the data might be due to the different case number with different immune stages involved in each study. Accumulating evidences indicated that TIPE2 might limit T cell responses during chronic viral infections [28]. In our study, TIPE2 might suppress HBV-mediated innate and adaptive immune response and contribute to the persistence of HBV infection. Furthermore, we reported that the level of TIPE2 mRNA in IC phase was significantly higher than IT phase; whereas the level of TIPE2 mRNA in ENH phase was also obviously higher than LR phase. These results suggested that immune-activated CHB patients (IC and ENH phases) had significantly higher TIPE2 levels than immune tolerant and/or inactive patients, and strongly supported the hypothesis that TIPE2 might participate in the activation of immune response in chronic HBV infection.

In this present study, we reported the elevated levels of IL-6, IL-10 and TNF-α in CHB patients compared with healthy controls. Several studies previously reported that TIPE2 could binds to caspase-8 and inhibit activating protein-1 and nuclear factor-kappaB(NF-κB) activation [9]. Meanwhile, it has been reported that the activated NF-κB can stimulate the expression of inflammatory cytokine [29], including TNF-α, IL-6, IL-1 and interferon-β [30]. In agreement with these reports, we demonstrated that the increased TIPE2 level was negatively correlated with IL-6, TNF-α and IFN-γ in CHB patients, and was positively correlated with IL-10. In addition, IL-10 has also been reported to be correlated with chronic progression of HBV infection and predict the prognosis of liver failure [31, 32]. Therefore, these results strongly suggested that TIPE2 might play an essential role in the T helper cellular immunity in the pathogenesis of HBV infection. Furthermore, we also revealed that the level of IL-10 in IC phase was significantly higher than that in LR or ENH phase, indicating that TIPE2 and IL-10 might exert important roles in the progression of IC from IT. However, the mechanism for TIPE2 in the regulation of immune clearance remains future research.

To testify the possible association of TIPE2 mRNA with HBV replication, we also investigated the TIPE2 mRNA levels stratified by HBeAg statue and HBV DNA. There were no significant differences in the levels of TIPE2 mRNA between HBeAg positive and negative patients and it was hypothesized that HBeAg might not be a crucial factor for regulating TIPE2 expression. However, we found a weak significant difference of TIPE2 in HBVDNA positive and negative group. Of note, we also demonstrated that HBV DNA was an independent positive factor for TIPE2 mRNA using multivariate analysis. These results might be explained by the phenomena that HBV DNA usually showed higher level in IC stage than ENH stage. In this present study, we provided the hypothesis that TIPE2 mRNA might be as a biomarker to discriminate IC phase from IT phase, and ENH phase from LR phase. We reported that a cut off values of 2.02 and 1.59 for the level of TIPE2 mRNA have significant power in discriminating IC from IT, and ENH for LR respectively. These results might provide a new diagnosis tool for early detecting IC phase from IT phase, and ENH phase from LR phase. However, a larger, multi-centered and prospective cohort should be established for validate this hypothesis.

Consistent with the notion that TIPE2 is a negative regulator for immune homeostasis, TIPE2 is a cytoplasmic protein preferentially in lymphoid tissues, especially highly in monocytes and T cells [33–35]. However, human TIPE2 was also expressed in a wide variety of non-hematopoietic cell types, including hepatocytes [35]. In this present study, we demonstrated that TIPE2 protein was mainly distributed in hepatocytes plasma in CHB patients. The expression of human TIPE2 in hepatocyte suggested that TIPE2 may be involved in maintaining immune privilege liking immune negative regulators [35]. We also reported that intrahepatic TIPE2 protein in inflammation G3/4 and fibrosis S3/4 was significantly increased compared to that of inflammation G3/4 and fibrosis S1/2. It could be explained by the fact that inflammation mainly involves in local hepatocytes and TIPE2 might participate in this progression.

There are also some limitations in the present study. First, we determined the TIPE2 mRNA and protein levels in relative small number of patients. In this study, we only included 29 liver tissue due to the fact that only a small number of patients accept liver biopsy in real world. Second, other HBV associated liver diseases including cirrhosis, acute hepatitis, and liver failure should be included to explore the comprehensive role of TIPE2 in the whole progression of HBV infection. In fact, there should be a series studies on this issue, we have recently reported the expression of TIPE2 in acute-on-chronic hepatitis B liver failure [27]. Third, our study is a cross-sectional cohort but not a perspective study; therefore, we did not include resolved HBV cases and patients with/after treatment. Finally, silence of TIPE2 gene in cell lines and HBV transgenic mice should be comprehensively investigated for the interplay of TIPE2 and HBV in the future.

In summary, TIPE2 might be associated with immune clearance of patients with chronic hepatitis B. Furthermore, the optional cut off values of 2.02 and 1.59 for TIPE2 mRNA as biomarker have strong power in identifying CHB patients with IC and ENH phases from patients with IT and LR phases.

## PATIENTS AND METHODS

### Patients

A total of 205 naïve treated CHB patients with CHB were enrolled in the Department of Hepatology, Qilu Hospital of Shandong University from Dec 2013 to Jan 2015. Of these patients, there were 25 CHB patients receiving liver biopsy. 15 healthy volunteers were included as health controls. Additionally, four liver specimens from healthy liver transplant donors serve as normal tissue. CHB were defined as the positivity of HBsAg for more than 6 months and no history of anti-HBV treatment within one year. IT phase was identified as HBeAg positive with a high virus load >5E+07 IU/mL and persistently normal ALT (n = 40). IC phase was identified as HBeAg positive with an elevated ALT and a virus load >2000 IU/mL(n = 100). LR phase was identified as HBeAg-negative with virus load < 2000 IU/mL and persistently normal ALT(n = 28). ENH phase was identified as HBeAg negative with virus load >2000 IU/mL and elevated ALT level(n = 37). The upper limit of normal of ALT was 40 U/L. Liver samples were histologically graded and staged according to the Scheuer scoring [36].

In addition, exclusive criteria are as the following: (1) infected with human immunodeficiency virus, hepatitis C virus, hepatitis D virus and autoimmune or metabolic liver disease;(2) had a treatment-free interval within one year; (3) hematologic disorders;(4) severe alcohol abuse. All the subjects have written informed consents which were approved by the local Ethic Committee at Qilu Hospital of Shandong University according to the Declaration of Helsinki [37].

### RNA and cDNA preparation from PBMC

5 ml of Natrium citrate-anticoagulated peripheral blood from CHB patients and heathy controls were collected. After Ficoll-Paque Plus (GE Healthcare, Uppsala, Sweden) density gradient centrifugation, peripheral blood mononuclear cells from the interface were collected and washed three times with phosphate-buffered saline. RNA was then extracted using TRIzol (Invitrogen, Carlsbad, CA, USA). 2 uL of RNA were reversely transcribed into cDNA using first-strand cDNA synthesis kit (Fermentas, Vilnius, Lithuania). Finally, the cDNA quantification was determined by reverse-transcriptase polymerase chain reaction (RT-PCR) method.

### Quantitative real-time PCR

The expression of TIPE2 in gene was examined by real-time PCR. β -actin was used as the endogenous control. Real-time PCR was conducted with Lightcycler 480 (Roche Diagnostics, Germany). Real-time PCR was preformed using a SYBR Premix Ex Taq™ (Takara, Japan) according to the manufacture's instructions in a total volume of 20 μl containing 1μlcDNA, 0.4μl specific primers, 10μl SYBR Green premix, and 8.2μl ddH2O. The primers sequences were list in Table 5. The reaction of PCR procedure was the following: denaturation at 95 °C for 30 s, 40 cycles at 95 °C for 5 s, 60 °C for 30 s and 72 °C for 30 s. Each sample was carried out in triplicate. LightCycler 480 Software (Roche Diagnostics, Germany) was used for data analysis, and the results were performed using the comparative (2 ^−^△△^Ct^) method.

**Table 5:** Primers sequence used for real-time RT-PCR

Gene	Primer sequence (5′-3′)
TIPE2	Forward GGAACATCCAAGGCAAGACTG
	Reverse AGCACCTCACTGCTTGTCTCATC
TNF-α	Forward AAGCCTGTAGCCCATGTTGT
	Reverse CAGATAGATGGGCTCATACC
IL-6	Forward ACCCCTGACCCAACCACAAAT
	Reverse AGCTGCGCAGAATGAGATGAGTT
IL-10	Forward ATGCTTCGAGATCTCCGAGA
	Reverse AAATCGATGACAGCGCCGTA
IFN-γ	Forward GCAGAGCCAAATTGTCTCCT
	Reverse ATGCTCTTCGACCTCGAAAC
β-actin	Forward ATGGGTCAGAAGGATTCCTATGTG
	Reverse CTTCATGAGGTAGTCAGTCAGGTC

### Immunohistochemistry staining

The intrahepatic expression of TIPE2 was determined using Polink-2 plus® Polymer HRP Detection System (Zhongshan Golden bridge Biotechnology, China). Briefly, the paraffin sections (5 μm) were deparaffinized using xylene and hydrated through graded ethanol (100%, 96%, 90%, and 70%) and distilled water. Endogenous peroxidase was blocked in 3% H_2_O_2_ for 15 minutes. After PBS washing, the slides were incubated using rabbit anti-TIPE2 polyclonal antibody (dilution 1:50, ProteinTech Group (Chicago, IL, USA) at 4°C for overnight and then polymer Helper or poly-HRP anti-rabbit IgG for 20 min at 37°C. After counterstaining with hematoxylin, the slides were dehydrated and mounted for viewing. Negative control was performed by same method of immunohistochemical staining except for replacing anti-TIPE2 polyclonal antibody with PBS. The immunostaining intensity were captured by a camera (OLYMPUS IX83, Tokyo, Japan) attached to the microscope. The Mean integrated optical density (integrated optical density sum/positive area sum) of all the diaminobenzidine-stained areas of each photo was measured by Image-Pro Plus 6.0 software (Media Cybernetics, Bethesda, MD).

### Clinical parameters

Blood samples were taken from each participant. The serum biochemical parameters (COBAS integra 800, Roche Diagnostics, Germany) included AST, ALT, total bilirubin (TBIL), albumin (ALB), and creatinine (Cr). Hemostasis parameters (ACL TOP 700, Instrument laboratory, USA) included PT-INR and PTA. WBC, HGB and PLT were also determined (Sysmex XE-2100, Sysmex Corporation, and Japan). Hepatitis B virus markers, including HBsAg, HBeAg, and anti-HBe were measured (cobas 6000 analyzer series, Roche Diagnostics, Switzerland).

### Statistical analysis

Data were shown as median values and interquartile ranges. Comparison between groups was performed by the Kruskal-Wallis analysis. Multivariate regression model and Spearman's tests were performed for correlation analyses. AUROC was used to determine the diagnostic accuracy. *P* < 0.05 was considered as statistical significance. All the statistical analyses were preformed using the IBM SPSS 19.0 software (SPSS Inc., Chicago, IL, USA).
